# Time-resolved colored petri net modeling of the eight extraordinary vessels in acupuncture

**DOI:** 10.1093/bib/bbaf631.056

**Published:** 2025-12-12

**Authors:** Quan Gan, Qi-Wei Ge, Zhengyang Qiu, Haoran Niu, Chuanxia Liu, Chen Li

**Affiliations:** School of Computer Engineering, Jiangsu Ocean University, Lianyungang, 222005, China; The Graduate School of East Asian Studies, Yamaguchi University, Yamaguchi, 753-8513, Japan; School of Computer Science and Technology, Beijing Jiaotong University, Beijing, 100044, China; School of Computer Engineering, Jiangsu Ocean University, Lianyungang, 222005, China; School of Foreign Languages, Jiangsu Ocean University, Lianyungang, 222005, China; Department of Human Genetics and Women's Hospital, Zhejiang University School of Medicine & Zhejiang Provincial Key Laboratory of Genetic and Developmental Disorders, Hangzhou, 310006, China

## Abstract

**Background:**

Acupuncture, a core therapy in Traditional Chinese Medicine (TCM), regulates physiological balance through intricate interactions along the meridian system. The Eight Extraordinary Vessels (EEVs) play a central role in redistributing Qi, Blood, and Body Fluids (QBF); however, many existing computational models overlook the physiological influence of acupuncture system and fail to incorporate its inherent time-scale regulatory mechanisms, thereby constraining the advancement of quantitative evaluation of acupuncture efficacy.

**Methods:**

We developed a time-resolved Colored Petri Net (CPN) framework that formalizes both the structural and dynamic aspects of acupuncture. The model integrates three key modules: (i) acupoint-meridian-organ coupling, encoding stimulation signals as tokenized inputs propagating through meridians; (ii) EEV-mediated transport, representing auxiliary QBF redistribution channels for systemic regulation; and (iii) dual-timescale drivers, capturing both session-level (characterized by latent, rising, peak, and waning phases within each 20–30 min treatment) and course-level (accumulate and attenuate effects over 10–15 days) to reproduce progressive therapeutic consolidation.

**Results:**

In a Parkinson’s disease-related insomnia case, the model simulated the acupuncture treatment process and reproduced organ recovery to healthy levels by Days 3–4, consistent with reported clinical timelines. The ablation study confirmed the essential roles of the EEV and dual-timescale modules, as removing either component led to slower convergence or loss of stability. In extended simulations, a duration sweep ranging from 1 to 15 days identified Day 7 as the optimal treatment window, effectively balancing therapeutic efficacy and patient burden.

**Conclusion:**

This study presents a quantitative CPN framework that integrates EEV-mediated regulation and dual-scale temporal dynamics in acupuncture. The model enhances interpretability by establishing a transparent mapping from clinical prescriptions to computationally derived organ-state trajectories. By providing a reproducible, quantitative, and testable framework, it advances the evidence-based modernization of acupuncture and lays the groundwork for broader computational approaches to investigate complex therapeutic mechanisms.

